# Exertional Desaturation Is More Severe in Idiopathic Pulmonary Fibrosis Than in Other Interstitial Lung Diseases

**DOI:** 10.1298/ptr.E10218

**Published:** 2023-02-14

**Authors:** Kohei OTAKE, Shogo MISU, Takashi FUJIKAWA, Hideki SAKAI, Hiromi TOMIOKA

**Affiliations:** ^1^Department of Rehabilitation, Kobe City Medical Center West Hospital, Japan; ^2^Department of Physical Therapy, Faculty of Nursing and Rehabilitation, Konan Women’s University, Japan; ^3^Department of Rehabilitation, Kobe City Medical Center General Hospital, Japan; ^4^Department of Respiratory Medicine, Kobe City Medical Center West Hospital, Japan

**Keywords:** Idiopathic pulmonary fibrosis, Interstitial lung disease, 6-minute walk test, Oxygen desaturation

## Abstract

Objective: Interstitial lung disease (ILD) is classified into several disease groups. Among them, idiopathic pulmonary fibrosis (IPF) has higher incidence and poor prognosis; therefore, it is important to characterize specific IPF symptoms. Exercise desaturation is a strong factor related to mortality in patients with ILD. Thus, the purpose of this study was to compare the degree of oxygen desaturation between IPF and other ILD (non-IPF ILD) patients during exercise, using the 6-minute walk test (6MWT). Methods: This retrospective study included 126 stable patients with ILD who underwent 6MWT in our outpatient department. The 6MWT was used to assess desaturation during exercise, 6-minute walk distance (6MWD), and dyspnea at the end of exercise. In addition, patient characteristics and pulmonary function test results were recorded. Results: Study subjects were divided into 51 IPF patients and 75 non-IPF ILD patients. The IPF group had significantly lower nadir oxygen saturation determined by pulse oximetry (SpO_2_) during 6MWT than the non-IPF ILD group (IPF, 86.5 ± 4.6%; non-IPF ILD, 88.7 ± 5.3%; *p* = 0.02). The significant association between the nadir SpO_2_ and IPF or non-IPF ILD grouping remained even after adjusting for gender, age, body mass index, lung function, 6MWD, and dyspnea (β = −1.62; *p* <0.05). Conclusion: Even after adjusting for confounding factors, IPF patients had lower nadir SpO_2_ during 6MWT. Early assessment of exercise desaturation using the 6MWT may be more important in patients with IPF compared with patients with other ILDs.

**I**nterstitial lung disease (ILD) comprises a group of diverse diseases that damage the lung parenchyma through inflammation and fibrosis. ILD is divided into several disease groups, with different treatment goals and assessment methods^1)^. Idiopathic pulmonary fibrosis (IPF) is the most frequent form of ILD and has a very poor prognosis with a median survival time of 2–3 years from diagnosis^2)^. In contrast, non-IPF ILDs, such as idiopathic nonspecific interstitial pneumonia (NSIP), connective tissue disease-associated ILD, and hypersensitivity pneumonitis, usually have a better prognosis than IPF^3)^. Hence, it is important to characterize the specific symptoms in IPF patients compared with those in non-IPF ILD patients.

Chronic hypoxemia influences prognosis in patients with ILD. Many patients with ILD present with severe desaturation during exercise even if they do not have hypoxemia at rest^4,5)^. The 6-minute walk test (6MWT) is an inexpensive and simple test that assesses desaturation during exercise and has been shown to be reproducible in patients with ILD^6,7)^. Desaturation during exercise is a strong risk factor for mortality in ILD patients^5,6,8–12)^ and is also associated with quality of life^13,14)^. A few studies have assessed the difference in desaturation during exercise between IPF patients and non-IPF ILD patients, but no adjustment was made for some important confounding factors^5,15)^. Moreover, several studies reported controversial results on whether desaturation during exercise is more severe in patients with IPF^16,17)^; hence, this relationship remains unclear. Clarifying the differences in desaturation during exercise between patients with IPF and patients with non-IPF ILD will be useful in determining how to evaluate IPF patients and help determine the appropriate treatment strategy.

Therefore, the purpose of this study was to compare the degree of oxygen desaturation in IPF and non-IPF ILD patients during exercise, using the 6MWT.

## Methods

### Study design and study population

This retrospective study included 195 stable patients with ILD who underwent the 6MWT as part of an examination at our outpatient clinic at Kobe City Medical Center West Hospital (Japan) between April 2016 and March 2020. No respiratory rehabilitation was performed before or after 6MWT because it was performed to assess physiological status of the patients. Among these, patients with long-term oxygen therapy, active coronary artery disease, other severe comorbidities, and missing data were excluded. The classification of ILD, including the diagnosis of IPF, was made using the American Thoracic Society/European Respiratory Society (ATS/ERS) consensus statement^1)^. Assessment items for the analyses were age, gender, the modified British Medical Research Council (mMRC) scale, comorbidities, treatment, smoking status, 6MWT, and pulmonary function tests.

This study was approved by the Institutional Review Board (Clinical Research) of Kobe City Medical Center West Hospital with waiver of informed consent because of its retrospective nature (June 25, 2019: project approval number 19-006).

### 6-minute walk test

The subjects performed 6MWT on a flat, 25-m walking course according to the ATS statement^18)^. Patients were instructed to walk as much as possible in 6 min. Oxygen saturation determined by pulse oximetry (SpO_2_) during 6MWT was continuously recorded using a pulse oximeter with a finger probe (WristOx 3150; Nonin Medical, Plymouth, MN, USA) and 6MWT analysis software (WristOx 2; Star Product, Tokyo, Japan). To minimize errors in SpO_2_ measurement, patients were instructed to hold the finger with the pulse oximeter attached to the front of their chest while walking. SpO_2_ was also measured at rest for 1 min prior to the test and for 3 min immediately after. Patients were asked to rate their dyspnea every minute during 6MWT, using the modified Borg scale scores by selecting a number from 0 to 10, with 0 being no appreciable dyspnea and 10 being maximal sustained dyspnea^19)^. From the 6MWT, 6-minute walk distance (6MWD), rest SpO_2_, nadir SpO_2_, ΔSpO_2_ (rest SpO_2_ − nadir SpO_2_), maximum heart rate, and modified Borg scale scores during 6MWT were obtained.

### Pulmonary function tests

All patients were subjected to pulmonary function tests by spirometry (Autospirometer System 7; MINATO, Osaka, Japan) according to the method described in the ATS 1994 update^20)^. Vital capacity (VC), forced vital capacity (FVC), and forced expiratory volume in 1 s (FEV_1_) were evaluated. Predicted normal values for the Japanese population were derived from reference values of the Japanese Respiratory Society^21)^ for VC, FVC, and FEV_1_.

### Analysis

Statistical analysis was performed with EZR Ver. 1.38 (Saitama Medical Center, Jichi Medical University, Saitama, Japan). Subjects were categorized into the IPF group or the non-IPF ILD group. Continuous variables were presented as mean ± standard deviation and were compared using unpaired t, Welch’s t, and Mann–Whitney U tests between the two groups. A simple regression analysis was performed to examine the relationship between the nadir SpO_2_ and IPF or non-IPF ILD groups. Additionally, multiple regression analysis was performed with the nadir SpO_2_ as the dependent variable and IPF diagnosis as the independent variable to evaluate the relationship after adjusting for potential covariates. Dummy variables were created with patients with IPF as 0 and non-IPF ILD as 1. These were entered into the regression analysis. Gender, age, body mass index (BMI), chronic obstructive pulmonary disease (COPD) or non-COPD, 6MWD, %VC, and mMRC scale scores were included as confounders because they are assumed to be involved in desaturation^22,23)^. *p*-value <0.05 was considered significant.

## Results

**Fig. 1.**
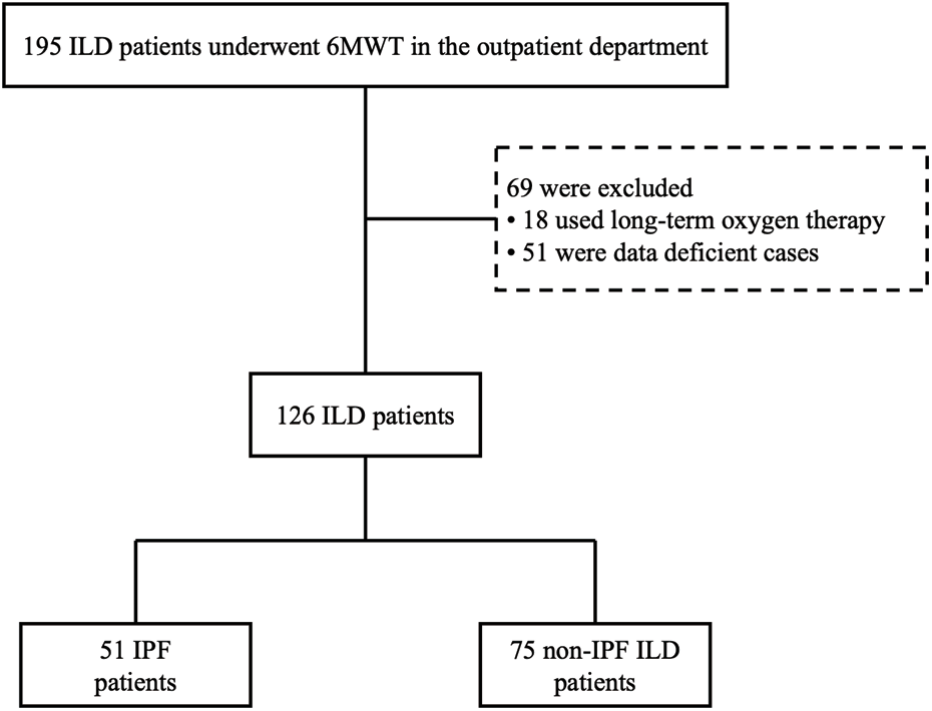
Flow chart showing exclusion and categorization of patients

ILD, interstitial lung disease; 6MWT, 6-minute walk test; IPF, idiopathic pulmonary fibrosis

Among 195 patients with ILD who underwent 6MWT, 18 patients were on long-term oxygen therapy and 51 cases had missing data. Therefore, 126 cases were included in the analysis. Fifty-one patients (40%) were diagnosed with IPF and the other 75 patients (60%) had non-IPF ILD. Non-IPF ILD included collagen disease, n = 12 (16.0%); hypersensitivity pneumonitis, n = 10 (13.3%); idiopathic pleuroparenchymal fibroelastosis, n = 6 (8.0%); asbestosis, n = 6 (8.0%); idiopathic NSIP, n = 3 (4.0%); sarcoidosis, n = 2 (2.7%); cryptogenic organizing pneumonia, n = 1 (1.3%); and unclassifiable, n = 35 (46.7%). Figure 1 shows the flow chart showing exclusion and categorization of patients.

Table 1 summarizes the patient characteristics according to the IPF or non-IPF ILD grouping. No significant differences were observed in %VC (IPF: 69.3% ± 16.3%, non-IPF ILD: 71.4% ± 17.5%; *p* = 0.50) and %FVC (IPF: 71.5% ± 18.6%, non-IPF ILD: 72.7% ± 18.6%; *p* = 0.73), between the IPF group and the non-IPF ILD group. In addition, no significant differences were observed in the other pulmonary function measures or patients characteristics between the two groups, with the exception of treatment modalities used for IPF and non-IPF ILD.

**Table 1.** Characteristics of patients according to the IPF or the non-IPF ILD grouping

Variables	IPF (n = 51)	non-IPF ILD (n = 75)	*p*-value
Gender (male), n (%)	37 (72.5)	48 (64.0)	0.42
Age (year)	72.9 ± 8.3	71.7 ± 9.4	0.47
BMI (kg/m^2^)	23.6 ± 3.6	23.1 ± 3.9	0.41
mMRC (0/1/2/3/4)	2/20/20/8/1	8/29/23/15/0	0.50
Smoking status			0.57
Current, n (%)	10 (19.6)	17 (22.7)	
Previous, n (%)	30 (58.8)	37 (49.3)	
Never, n (%)	11 (21.6)	21 (28.0)	
Comorbidities, n (%)			
Hypertension	12 (23.5)	21 (28.0)	0.72
Hyperlipidemia	5 (9.8)	8 (10.7)	1.00
Diabetes	13 (25.5)	15 (20.0)	0.61
COPD	5 (9.8)	7 (9.3)	1.00
Lung cancer	5 (9.8)	7 (9.3)	1.00
Treatment, n (%)			
Corticosteroid	0 (0)	11 (14.7)	0.01
Immunosuppressive therapy	0 (0)	6 (8.0)	0.10
Anti-fibrotic therapy	8 (15.7)	0 (0)	0.002
Pulmonary function tests			
VC (L)	2.2 ± 0.6	2.2 ± 0.8	0.49
VC (%pred)	69.3 ± 16.3	71.4 ± 17.5	0.50
FVC (L)	2.1 ± 0.7	2.2 ± 0.8	0.46
FVC (%pred)	71.5 ± 18.6	72.7 ± 18.6	0.73
FEV_1_ (L)	1.8 ± 0.5	1.8 ± 0.6	0.80
FEV_1_ (%pred)	75.3 ± 18.9	74.9 ± 17.7	0.91
Data presented as n (%), or mean ± standard deviation			
IPF, idiopathic pulmonary fibrosis; non-IPF ILD, interstitial lung disease other than IPF; BMI, body mass index; mMRC, modified British Medical Research Council; COPD, chronic obstructive pulmonary disease; VC, vital capacity; FVC, forced vital capacity; FEV_1_, forced expiratory volume in 1 s			

Table 2 presents the results of the 6MWT according to the IPF or the non-IPF ILD grouping. The IPF group had significantly lower nadir SpO_2_ during 6MWT than the non-IPF ILD group (IPF: 86.5% ± 4.6%, non-IPF ILD: 88.7% ± 5.3%; *p* = 0.02). The IPF group also had significantly higher ΔSpO_2_ during 6MWT than the non-IPF ILD group (IPF: 9.3% ± 4.0%, non-IPF ILD: 6.9% ± 4.4%; *p* = 0.002). In contrast, no significant difference was observed in the dyspnea scale at the end of 6MWT (IPF, 2 [range, 0–10]; non-IPF ILD, 2 [range, 0–10]; *p* = 0.49) and the 6MWD (IPF: 410.6 ± 116.8 m, non-IPF ILD: 408.9 ± 119.6 m; *p* = 0.94) between the two groups.

**Table 2.** Results of the 6MWT according to IPF or non-IPF ILD grouping

Variables	IPF (n = 51)	non-IPF ILD (n = 75)	*p*-value
6MWD (m)	410.6 ± 116.8	408.9 ± 119.6	0.94
Rest SpO_2_ (%)	95.7 ± 1.3	95.6 ± 1.9	0.61
Nadir SpO_2_ (%)	86.5 ± 4.6	88.7 ± 5.3	0.02
ΔSpO_2_ (%)	9.3 ± 4.0	6.9 ± 4.4	0.002
Maximum pulse rate (bpm)	114.8 ± 15.8	110.9 ± 16.1	0.19
Modified Borg scale score*	2(0–10)	2(0–10)	0.49
Data are presented as mean ± standard deviation			
*The modified Borg scale score at the end of 6MWT			
6MWT, 6-minute walk test; IPF, idiopathic pulmonary fibrosis; non-IPF ILD, interstitial lung disease other than IPF; 6MWD, 6-minute walk distance; SpO_2_, oxygen saturation of determined by pulse oximetry; ΔSpO_2_, difference in SpO_2_ rest to nadir			

**Fig. 2.**
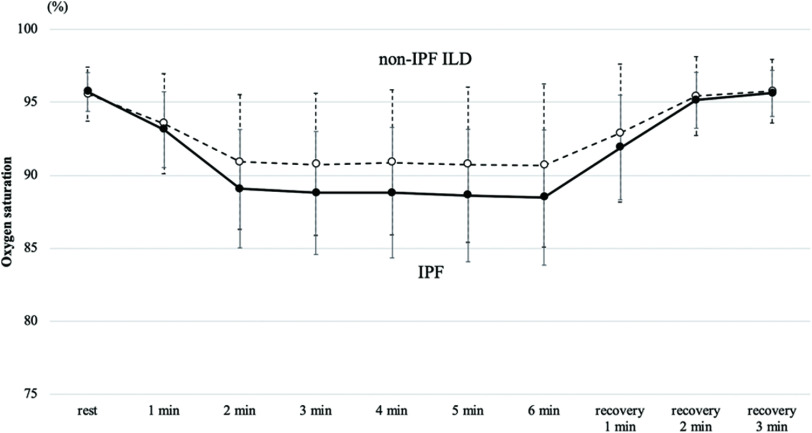
Mean SpO_2_ of the point reached for each minute during the 6-min walk test (solid line, IPF; dotted line, non-IPF ILD)

Figure 2 shows the change in SpO_2_ over time during the 6MWT.

Simple regression analysis revealed a significant association (B = −2.20, *p* = 0.02) between nadir SpO_2_ and IPF or non-IPF ILD grouping (Table 3: Model 1). The significant association (B = −1.62, *p* <0.05) remained even after adjustments for gender, age, BMI, COPD or non-COPD, 6MWD, %VC, and mMRC scale score (Table 3: Model 2).

**Table 3.** Univariate and multivariate analyses of association between nadir SpO_2_ and IPF or non-IPF ILD

	SpO_2_ (nadir) at 6MWT							
Variables	Model 1			Model 2				
	B	95% CI	*p*-value	B	β	95% CI	*p*-value	VIF
IPF or non-IPF ILD	−2.20	−3.99 to −0.41	0.02	−1.62	−0.14	−3.21 to −0.03	<0.05	1.03
Gender (male or female)				1.55	0.14	−0.18 to –3.27	0.08	1.10
Age (year)				−0.07	−0.13	−0.18 to –0.03	0.20	1.49
BMI (kg/m^2^)				−0.36	−0.27	−0.58 to −0.15	0.001	1.10
COPD or non-COPD				−3.65	−0.21	−6.51 to −0.79	0.01	1.19
6MWD (m)				−0.01	−0.22	−0.02 to −0.00	0.04	1.88
%VC				0.11	0.39	0.07 to –0.17	<0.001	1.23
mMRC^†^				−1.13	−0.20	−2.09 to −0.16	0.02	1.26
Model 1. Results of simple regression analysis								
Model 2. Results of multiple regression analysis with nadir SpO_2_ at 6MWT as the objective variable, IPF or non-IPF ILD as the explanatory variable, and gender, age, BMI, COPD or non-COPD, 6MWD, %VC, and dyspnea as confounding factors								
^†^Dyspnea was assessed with the mMRC scale.								
SpO_2_, oxygen saturation of determined by pulse oximetry; IPF, idiopathic pulmonary fibrosis; non-IPF ILD, interstitial lung disease other than IPF; 6MWT, 6-minute walk test; CI, confidence interval; VIF, variance inflation factor; BMI, body mass index; COPD, chronic obstructive pulmonary disease; 6MWD, 6-minute walk distance; VC, vital capacity; mMRC, modified British Medical Research Council								

non-IPF ILD, interstitial lung disease other than IPF; IPF, idiopathic pulmonary fibrosis; SpO_2_, oxygen saturation determined by pulse oximetry

## Discussion

In this study, we used the 6MWT to characterize the severity of desaturation during exercise in ILD patients. The results demonstrated that IPF patients had significantly lower nadir SpO_2_ during 6MWT than non-IPF ILD patients, although there was no difference in dyspnea scale results and 6MWD. In addition, even after adjusting for various potential confounding factors such as gender, age, BMI, COPD or non-COPD, 6MWD, %VC, and mMRC scale scores, IPF patients had lower nadir SpO_2_ during 6MWT. Therefore, desaturation during exercise can be severe in IPF patients regardless of dyspnea, physical function, lung capacity, and other factors.

The results of this study indicate that patients with IPF tended to have severe desaturation during exercise despite not having hypoxemia at rest. Holland et al. reported that nadir SpO_2_ in 6MWT was significantly lower in the IPF group than that in the non-IPF ILD group^15)^. Their finding supports our results, but the study did not adjust for any confounding factors. Another study comparing SpO_2_ during 6MWT between patients with IPF and patients with idiopathic NSIP reported that there was no significant difference^5)^. Although there were only two cases of idiopathic NSIP in the present study, the results could not be simply compared, and it is necessary to classify non-IPF ILD patients in detail and compare the results using 6MWT in the future.

We performed the 6MWT to assess desaturation during exercise in patients with ILD. In two previous studies, wherein a bicycle ergometer was employed as an exercise stress test, IPF patients were shown to exhibit more severe desaturation during exercise than non-IPF ILD patients^16,17)^. Although these results were similar to our findings, 6MWT is a more clinically feasible and simpler exercise stress test than using a bicycle ergometer. In addition, they also did not adjust for confounding factors. Therefore, this study expanded on the results of these two studies and indicated that similar results can also be obtained with 6MWT, which is simple and easy to administer in the clinical settings.

More severe desaturation during exercise in patients with IPF might be explained by more severe diffusion limitation. IPF is a progressive disease, and diffusing capacity for carbon monoxide (DL_CO_) is more likely to decline earlier compared with %FVC. In fact, it was reported that patients with IPF had lower DL_CO_ than patients with non-IPF ILD^17,24)^. Ventilation–diffusion inequality and diffusion limitation play an important role in desaturation at rest and during exercise^25)^. A number of studies showed that low DL_CO_ is strongly associated with desaturation during exercise. For example, Du Plessis et al. reported that DL_CO_ was a predictor of nadir SpO_2_ during 6MWT among ILD patients^22)^. In another study of 300 patients with ILD, a simple regression analysis identified DL_CO_ as the explanatory factor for decreased oxygen saturation during exercise^26)^. Therefore, we speculated that early decline in DL_CO_ in IPF patients could lead to more severe desaturation during exercise. However, this study did not have sufficient data on DL_CO_, which is a future challenge.

Desaturation during exercise is a strong prognostic indicator in patients with IPF^8,9)^. Our results indicated that IPF patients have more severe exercise desaturation compared with non-IPF ILD patients. Therefore, early assessment of exercise desaturation using 6MWT may be useful, especially in patients with IPF who do not have hypoxemia at rest. In addition, the 6MWT does not require much time or complex equipment to implement; therefore, the test is easy to perform clinically.

Our study has some limitations. First, this was a single-center cross-sectional study and may not represent the general characteristics of IPF patients. Therefore, further multicenter studies with larger samples are needed. Second, we did not evaluate the presence of pulmonary hypertension, right heart pressure, or other circulatory dynamics. It has been reported that patients with pulmonary hypertension present with severe exercise hypoxia^27,28)^. For that reason, these can be confounding factors. However, in the present study, there was no difference in the maximum pulse rate during the 6MWT, which is related to the circulatory response during exercise, so the difference in circulatory dynamics may not have been significant between IPF and non-IPF ILD patients.

## Conclusion

The results of this study showed that even after adjusting for confounding factors, IPF patients had lower nadir SpO_2_ during 6MWT. Early assessment of exercise desaturation using the 6MWT might be more important in patients with IPF than in patients with non-IPF ILD. Even if the resting SpO_2_ is good, it may be important to perform 6MWT more aggressively in patients with IPF to detect a decrease in oxygen saturation during the test, which is a prognostic factor at an early stage.

## Acknowledgments

The authors would like to thank Kiyohiko Kado, Chisato Nagatani, Takashi Kadooka, Shoko Mitani, Takuya Okamoto, Kumi Matumura, Ryoya Fujisaki, Yuya Kawate, Suzuka Higashiyama, Takumi Yamaguchi, Yutaro Oki, Yukari Oki, and Haruko Yamamoto for cooperation in acquisition of data and constructive comments on this paper. The authors would also like to thank Enago (www.enago.jp) for the English language review.

## Conflict of Interest

There is no conflict of interest to disclose.
